# Atomic-scale Studies of Uranium Oxidation and Corrosion by Water Vapour

**DOI:** 10.1038/srep25618

**Published:** 2016-07-12

**Authors:** T. L. Martin, C. Coe, P. A. J. Bagot, P. Morrall, G. D. W Smith, T. Scott, M. P. Moody

**Affiliations:** 1 University of Oxford, Department of Materials, Oxford, United Kingdom OX1 3PH; 2 AWE, Aldermaston, Reading, Berkshire, United Kingdom, RG7 4PR; 3 Interface Analysis Centre, University of Bristol, H.H. Wills Physics Laboratory, Bristol, United Kingdom, BS2 1TL

## Abstract

Understanding the corrosion of uranium is important for its safe, long-term storage. Uranium metal corrodes rapidly in air, but the exact mechanism remains subject to debate. Atom Probe Tomography was used to investigate the surface microstructure of metallic depleted uranium specimens following polishing and exposure to moist air. A complex, corrugated metal-oxide interface was observed, with approximately 60 at.% oxygen content within the oxide. Interestingly, a very thin (~5 nm) interfacial layer of uranium hydride was observed at the oxide-metal interface. Exposure to deuterated water vapour produced an equivalent deuteride signal at the metal-oxide interface, confirming the hydride as originating via the water vapour oxidation mechanism. Hydroxide ions were detected uniformly throughout the oxide, yet showed reduced prominence at the metal interface. These results support a proposed mechanism for the oxidation of uranium in water vapour environments where the transport of hydroxyl species and the formation of hydride are key to understanding the observed behaviour.

Uranium metal and its oxides have become synonymous with nuclear energy applications. Metallic uranium was used as the fuel material in the world’s first fission reactors but was subsequently replaced by uranium dioxide (UO_2_), which now forms the basis for fuel within the current generation of fission power reactors. Due to its inherently reactive and pyrophoric nature, legacy uranium remaining from first generation nuclear reactors now poses a significant challenge for both safe long-term storage and future disposal. Consequently understanding the mechanism by which uranium reacts with moisture at the atomic scale is essential for underpinning predictions of the ageing of stored legacy materials and informing the design of future storage and waste handling facilities. Insights into the corrosion mechanism of uranium at the atomic scale will be of great benefit to varied applications of uranium including hydrogen storage, neutron shielding and future high density nuclear fuel materials, as well as advancing the understanding of corrosion in a wide-range of metal systems, especially the similarly behaved actinide and lanthanide metals such as Ce, La, Gd, Th and Pu.

The underlying corrosion mechanism of uranium in the presence of water vapour and/or oxygen, has been a longstanding topic of debate^1^,^2^,^3^,^4^,^5^,^6^,^7^,^8^. Although anionic diffusion is agreed upon, several reaction mechanisms have been proposed with different dominant diffusing species, either OH^− ^1,2,3, O^2− ^4,5 or a combination^6^. In some cases it is suggested that uranium hydride is formed during the reaction^1^,^2^,^7^,^8^ as part of several proposed mechanisms: (1) as a product of reaction between uranium metal and free hydrogen (a known gas phase by-product of the oxidation of uranium by water); (2) as a possible intermediate (*e.g.* transition state) in the oxidation of uranium by hydroxyl species; or (3) that both hydride and oxide form as competing products of the hydroxyl oxidation mechanism. However, throughout the earlier literature there is no clear explanation of the morphology of this hydride in relation to the oxide being generated. Previous investigations into the presence and location of hydrides within the uranium corrosion process using techniques such as Secondary Ion Mass Spectroscopy (SIMS), have been limited by spatial resolution, the possibility of matrix effects (which influence the secondary ion intensity at the oxide-metal interface) and by the fact that the topography of the interface is uneven, preventing an accurate characterisation should thin layers of hydride exist^6^,^9^. If the presence of hydride, whether as an intermediary layer or isolated features, could be conclusively demonstrated it would greatly improve the understanding of the corrosion mechanism of uranium in moist air and water vapour environments. Such mechanistic understanding is needed for the construction of predictive corrosion models, as with the ionic diffusion model used to represent the oxidation of uranium in pure oxygen environments^10^. Unlike the application of empirical models to assess corrosion during long-term storage, such models allow insight beyond the limitations of the data used to generate the model.

Atom probe tomography (APT) is a 3D atomic scale microscopy technique suited to the study of nanoscale structures, interfaces and elemental gradients in materials. Advances in the technique^11^, particularly the introduction of laser-pulsing^12^,^13^ and the development of advanced Focused Ion Beam (FIB) techniques for specimen preparation^14^, have enabled the application of APT to a wider range of less conductive and/or brittle materials. APT has found an increasing number of applications in the investigation of metal-oxide systems^15^,^16^,^17^,^18^,^19^, including recently on uranium oxide^20^. The combination of high spatial resolution and elemental sensitivity make APT one of the only techniques suitable to identify localized or layered hydride should it form during uranium oxidation. To date, there has been no APT investigation of the surface microstructure of uranium during the early stages of water vapour corrosion, although there have been some investigations of uranium binary alloys in the late 1980s^21^,^22^ and limited investigations into uranium-niobium alloys using modern instruments^23^. This study aims to utilize the advances in APT instrumentation and preparation techniques over the past decade to investigate the oxide-metal interface of uranium exposed to moist air under ambient conditions and clarify the mechanism of water-driven uranium corrosion by unambiguously determining, or refuting, the presence and location of hydride within the corrosion product formed.

## Results

### Atom probe tomography of uranium exposed to air

APT utilises a needle-shaped specimen with a tip radius of between 20 nm and 100 nm. The technique exploits the effect of an intense electric field upon which is superimposed an ultrafast voltage or laser pulse, for the highly controlled field evaporation of individual ions from the surface of the specimen. Each detected ion can be directly correlated to the pulse which caused its evaporation from the tip, enabling measurement of the time-of-flight, and hence the chemical identification of the (molecular) ion. From the position at which each ion strikes the detector and the sequence of evaporation, a straightforward reverse-projection algorithm can be used to precisely reconstruct the original location in 3-dimensions (3D) within the specimen. Ultimately, a 3D atom-by-atom reconstruction of the specimen incorporating tens-to-hundreds of millions of atoms is generated, enabling high resolution elemental mapping.

In this study, metallic depleted uranium was mechanically polished to remove the pre-existing oxide. It was then exposed to air under ambient temperature and humidity conditions for one hour, immediately prior to FIB “lift-out” and polishing into needle specimens suitable for atom probe analysis.

The mass spectrum of uranium and uranium oxides consists broadly of three regions, which are shown in detail in the mass spectra shown in Figures S.1, S.2 and S.3 of the supplemental material. First, as shown in Figure S.1(a), is a region at 235 Da – 290 Da corresponding to the singly charged U isotope and its corresponding oxides and hydrides. Typically this signal is lower in intensity than the other two charge states, and the metal ion is less commonly found in this configuration.

The doubly charged region of the mass spectrum between 115 Da and 140 Da is shown in Figure S.2(a) of the supplemental material. This is the highest intensity region of the mass spectrum, with typically two orders of magnitude higher signal than the singular charge state peaks. The U_238_^2+^ peak at 119 Da is the dominant peak, with a smaller peak corresponding to the U_235_ isotope present at 117.5 Da. Both peaks have a single hydride peak at 118 Da and 119.5 Da respectively, and oxide and dioxide complex ions are observed for the U_238_ isotope at 127 Da and 135 Da. Small hydroxide and carbide peaks are also distinguishable.

A triple charged state is also observable, as shown in Figure S.3(a) in the supplemental information, with peaks at 78.33 Da and 79.33 Da, together with smaller peaks for UO and UO_2_. The intensity of the 3 + peaks is between that of the two of the other regions, but significantly all three regions have significantly higher intensity than Ga, as discussed further in the Method section.

**Figure 1:**
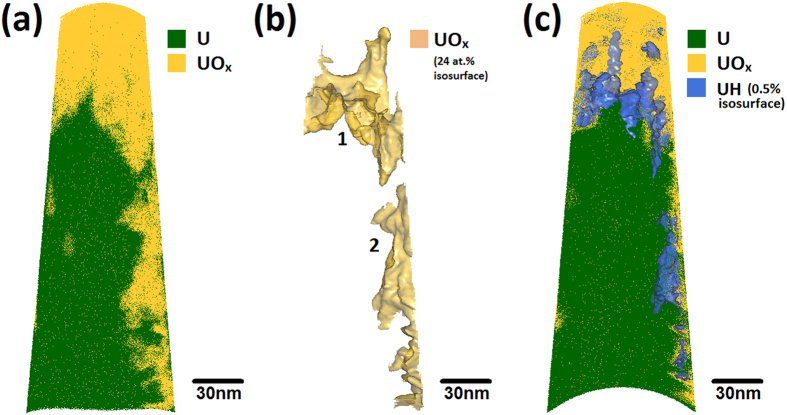
(**a**) An atom probe map of a tip extracted from a uranium sample exposed to air for approximately 1 hour, showing U and UO_x_ ions in green and yellow/orange, respectively. The original surface of the uranium is located at the top of the specimen. (**b**) A 24 at.% UO/UO_2_ isosurface indicating two oxide regions on the specimen; (marked 1) at the original surface and (marked 2) generated on the side of the specimen during sample preparation. (**c**) The same atom map as in (**a**), but with an isoconcentration surface indicating 0.5 at.% UH in blue to reveal the locations where hydride ions are detected. For (**c**), the front face of the dataset is cropped away to show a cross-section of the middle of the specimen.

Figure 1(a) shows a typical APT reconstruction for a uranium tip, featuring a surface-oxidised region of the material followed by the subsurface (unreacted) bulk metal region. The morphology of the oxide-metal interface was uneven and undulated in all specimens studied.

In Fig. 1(b) a 24 at.% UO_x_ isoconcentration surface is used to define the position of the oxide-metal interface. UO_x_ is defined as any complex ion containing both uranium and oxygen regardless of stoichiometry, whilst the 24% value was chosen as it gave the most defined interface shape. Figure 1(b) also indicates the presence of two distinct oxide features; the surface oxide marked as 1 and an incidental oxide feature marked as 2, where incidental is defined as the oxidation which occurred unintentionally due to atmospheric exposure during transportation of the tip to the atom probe instrument. An apparent increase in uranium hydride content is observed at the oxide-metal interface, as highlighted by the light blue 0.5 at.% UH isosurface plotted in Fig. 1(c).

**Figure 2:**
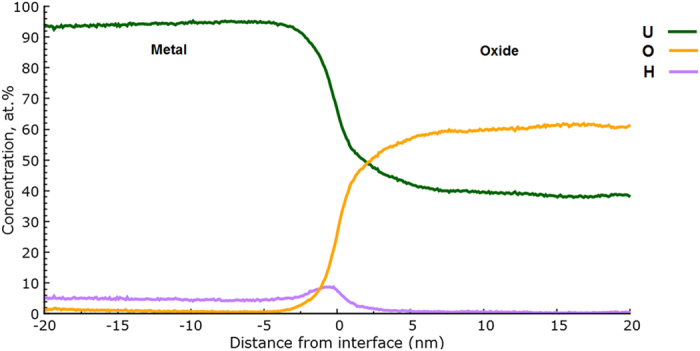
Proximity histogram of the surface oxide feature marked as 1 in Fig. 1(b). All complex ions are decomposed into their constituent elements.

The change in chemical composition across the surface metal-oxide interface labelled ‘1’ in Fig. 1(b) has been characterised using a proximity histogram (proxigram) analysis, shown in Fig. 2. Having defined a 3D surface, in this case using the UO_x_ isoconcentration surface in Fig. 1(b), the proxigram measures the chemical composition as a function of the perpendicular distance from this 3D surface^24^. Due to the tendency for complex ions of oxides to vary in oxidation state depending on where they are observed on the tip, Fig. 2 decomposes the proximity histogram into individual elemental contributions rather than complex ions. The decomposed profiles show a concentration of approximately 95 at.% U in the bulk metal, accompanied by small amounts of hydrogen and oxygen. Near the oxide-metal interface the oxygen content rises rapidly from virtually zero to around 50 at.% over a distance normal to the defined interface of approximately 3 nm. At around 5 nm above the interface the oxygen content plateaus at approximately 60 at.% oxygen. This slightly hypostoichiometric (*i.e.* UO_2−x_) ratio is consistent with Baker *et al*.^1^,^2^ who report oxidation involving water vapour as producing oxides of lower stoichiometry than the hyperstoichiometric oxides (*i.e.* UO_2+x_) formed in pure oxygen. It is important, however, to be cautious in overinterpreting such deficiencies in expected oxide chemistries as in APT there is a known dependence of oxide stoichiometry on laser energy^25^.

Most interestingly, the data demonstrates there is a significant increase in the hydrogen content at the interface between the metal and oxide regions. This hydrogen signal is fully accounted for from hydrogen combined within the uranium hydride molecular ions observed at 119.5 Da (UH^2+^) and 239 Da (UH^+^). The initial assignment of UH^+^ molecular ions as from uranium hydride, which has the formal stoichiometry of UH_3_, is substantiated using existing time-of-flight (ToF) SIMS and density functional theory (DFT) evidence^9^. The DFT suggests that molecular UH_x_ cations where x > 1 suffer from instability and are thus expected to decompose within the time-frame of ToF-SIMS measurements (which are comparable to APT measurement time-frames). The ToF-SIMS confirms that only UH^+^ cations are evidenced, while heavier molecular ions are observed only as anions (*i.e.* UH_x_^−^, where x ≥ 2). Anions of this type are not observed in APT because of the strongly positive electric field that is applied to the specimens.

All specimens which permitted observation of the oxide-metal interface also revealed evidence of increased UH content at that interface. In each case, this layer was measured at between 3 nm and 5 nm thick, with a similar profile to that observed in Fig. 2, and revealing peak concentrations of UH molecular ions varying between 12 at.% and 20 at.%. This value of the hydrogen content should be considered as a lower limit due to the instability of the higher order hydrides and since the signal from isolated H ions (*i.e.* those not detected as uranium containing molecular ions) were not included, due to the hydrogen contamination which is commonly present within stainless steel UHV systems (this is discussed in more detail below). The stoichiometry of the hydride is expected to be close to UH_3_. Although the oxide layer contained no signal from hydride molecular ions, other than at the interface, around 5 at.% H from UH ions was observed in the bulk metal matrix. This is certainly an artefact due to hydrogen contamination occurring from the analysis chamber during the time-frame of the experimental measurement. This effect is most noticeable in the metal, rather than the oxide, due to the increased reactivity of the fresh metal surface as it is systematically exposed during successive APT tip profiling measurements, and the fact that after the H is adsorbed, the UH ions are more readily evaporated than U. However, since APT does not suffer from the matrix effects which can limit the reliability of SIMS measurements at interfaces, one must conclude that the increase in the hydride molecular ion intensity at the oxide-metal interface is strongly indicative of the presence of a genuine discrete hydride layer, since its intensity is considerably higher than the 5 at.% background contamination evidenced within the bulk metal.

**Figure 3:**
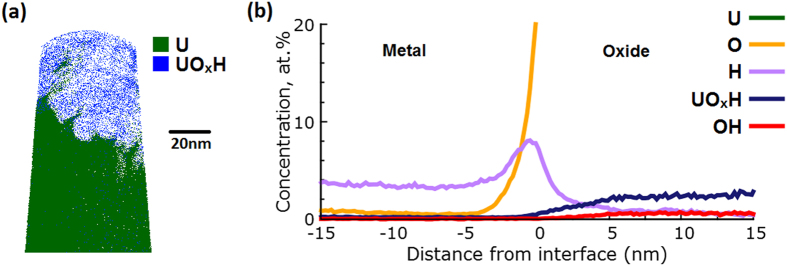
(**a**) atom map showing the location of uranium hydroxide ions in the same dataset shown in Figs 1 and 2(b) a magnification of the proxigram across the metal-oxide interface for the same specimen, showing the distribution of UO_x_H and OH species within the oxide. The front of the dataset has been cropped to better show the internal structure of the oxide-hydride-metal interface.

The evidence of a hydride layer forming at the oxide-metal interface clearly suggests the involvement of hydroxyl ions in the oxidation mechanism, but does not prove it. Figure 3(a) shows an atom map which reveals the location of hydroxyl species, together with a proxigram similar to that in Fig. 2, but with the OH-related complex ion species ranged as separate signals to clarify their position relative to the decomposed O and H signals. The dataset has also been rotated and cropped at the front compared to Fig. 2, to better illustrate the position of the U_x_OH ions. The proxigram data in Fig. 3(b) indicates hydroxyl content within the corrosion layer; from evidence at 17 Da (OH^+^), 127.5 Da (UOH^2+^), 255 Da (UOH^+^), 135.5 Da (UO_2_H^2+^) and 271 Da (UO_2_H^+^). This hydroxyl content is evenly distributed throughout the oxide, but is absent at the interface with the metal where the hydride signal is observed, suggesting that the hydroxyl species decompose at the hydride layer. This result agrees well with the basic mechanism proposed by Baker in which both hydride and oxide are formed from hydroxyl species which diffuse through the oxide^1^,^2^.

It is difficult to be completely confident about the origin of the hydride features due to the experimental limitations of current atom probe instruments with respect to hydrogen analysis^26^. Even within the ultra-high vacuum (UHV) of the LEAP instrument (around 2 × 10^−11^ Torr), significant quantities of hydrogen in the form of water, hydrocarbons and hydrogen molecules exist on the analysis chamber walls, sample and in the gas phase. In order to conclusively demonstrate the existence of the interfacial hydride layer, we used an innovative corrosion method employing deuterated water, which offers the unambiguous determination of deuterium and deuteride species. Clearly, due to the higher mass/charge ratio of deuterium compared to natural hydrogen, these deuterated species are distinct from the hydrogen and hydride species present due to contamination from within the atom probe analysis chamber.

### Atom probe tomography of uranium exposed to deuterated water vapour

Using deuterium provides an unambiguous signal to locate where the hydrogen ultimately resides in a material since it does not naturally occur in significant quantities and so any D-containing peaks in the mass spectrum can be positively attributed to the corrosion mechanism being tested^27^,^28^. Charging a material like steel with deuterium, then preparing it for later deuterium analysis can be very challenging due to the high permeability of H in Fe, requiring cryogenic cooling of the sample immediately after charging to ensure the deuterium signal is not lost prior to analysis^28^. However, compared to the difficulty associated with observing highly mobile, soluble hydrogen within a metal matrix the chemical stability of a hydride phase vastly improves its probability of detection. Thus, this study uses exposure to deuterated water (D_2_O) to unambiguously identify the involvement and location of any hydride formed during the water vapour oxidation of uranium.

**Figure 4:**
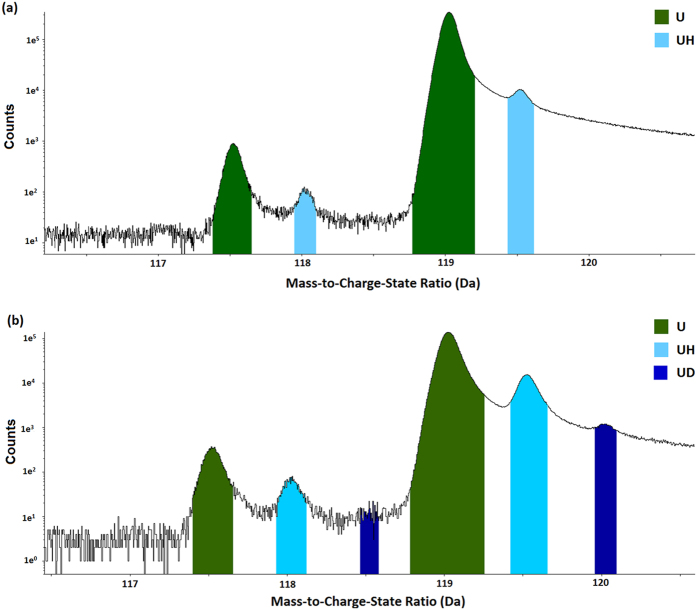
Comparison of the mass spectra (in log scale) magnified to concentrate on the U^2+^ ion and its respective hydrides and deuterides summed across the entire specimen for (**a**) the depleted uranium sample exposed to air under ambient conditions for one hour as shown in Fig. 1(b) the uranium sample exposed to D_2_O vapour as shown in Fig. 5.

Figure 4 compares the mass spectra for the two experiments, specifically the difference between the U^2+^ ion and its hydrides and deuterides. This information is also replicated for the entire mass spectra in Figures S.1(b), S.2(b) and S.3(c) of the supplemental information. For the comparison of the hydride behaviour between the ambient and deuterated cases in Fig. 4, we concentrated our analysis on the U^2+^ case, where due to the higher intensity the hydride peaks are much clearer and easier to interpret than for the U^+^ or U^3+^ ions.

In the specimen exposed to air with ambient humidity, the ^235^U and ^238^U ions are observed at 117.5 Da and 119 Da, respectively, as well as the monohydride complex ions at 118.0 Da and 119.5 Da. For the uranium exposed to D_2_O vapour, a distinct peak is observed at 120.0 Da that is not present in the previous spectra, which we attribute to uranium deuteride. In addition, there is a small correlated feature at 118.5 Da that is associated with ^235^UD. This is a clear indication that despite several weeks of storage under inert gas between the preparation and analysis of these specimens, some deuterium remains in the material. This finding necessitates that the deuterium is chemically bound, and therefore further supports our assignment of the feature to be UD_3_ (the deuterated uranium hydride).

**Figure 5:**
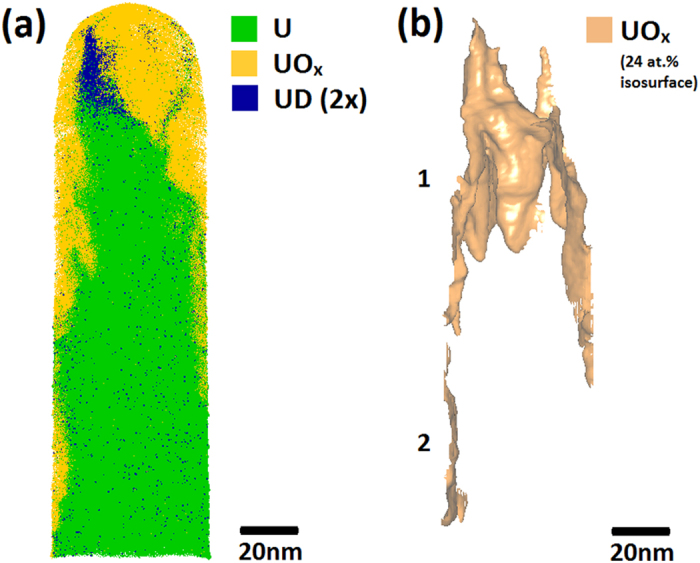
(**a**) Atom map for the uranium sample exposed to deuterated water for approximately 2 hours, highlighting the region of uranium deuteride. The original surface of the sample is located at the top of the specimen. The uranium deuteride molecular ions (in blue) are displayed at twice the size of the uranium and uranium oxide ions to clarify their position near to the oxide-metal interface. Figure 5(b) shows the 24 at.% UO/UO_2_ isosurface used to calculate the proximity histograms in Fig. 6.

Figure 5 shows the atom map for a uranium specimen exposed to D_2_O vapour. A corrugated oxide structure similar to that shown in Fig. 1 is observed. As before, a 24 at.% isosurface of both UO and UO_2_ complex ions has been created and shown in Fig. 5(b), with each distinct region labelled. Here we again see two distinct features; the surface oxide marked as 1 and an incidental oxide (defined again as the oxidation occurring due to atmospheric exposure during transportation of the tip to the atom probe instrument) marked as 2.

Figure 5(a) highlights the layer of uranium deuteride formed as part of the oxidation reaction with D_2_O. The location of the deuteride is equivalent to that of the hydride revealed in Fig. 1. However, in these deuterium-labelled oxidation experiments, the deuteride at the oxide-metal interface can only have formed from the reaction with D_2_O. This experiment therefore confirms the proposed hydroxyl driven mechanism by removing any possibility of an experimental artefact being responsible for the observed hydride layer.

We note that when displaying the position of UD atoms in the dataset shown in Fig. 5a, it is not possible to distinguish between those atoms belonging to actual UD signal and those due to the thermal tail of the nearby (and much larger) 119 Da U peak, even though the presence of the UD peak above this thermal tail background is clear and unambiguous in the mass spectrum shown in Fig. 4b. As it is not possible to distinguish these contributions on an atom-by-atom basis, there will always be some ions labelled as UD further down the shank that have a mass/charge ratio of 120, as seen in the dark blue dots towards the bottom of the dataset in Fig. 5a. These thermal tail contributions are randomly distributed, whereas significantly there is a large concentration of 120 Da ions at the interface of the oxide and metal, and so we ascribe the concentration of ions at the interface to be UD, and the randomly distributed ions as remnants of the 119 Da U thermal tail. Both are shown in Fig. 5a to give a true representation of the 120 Da peak location.

**Figure 6:**
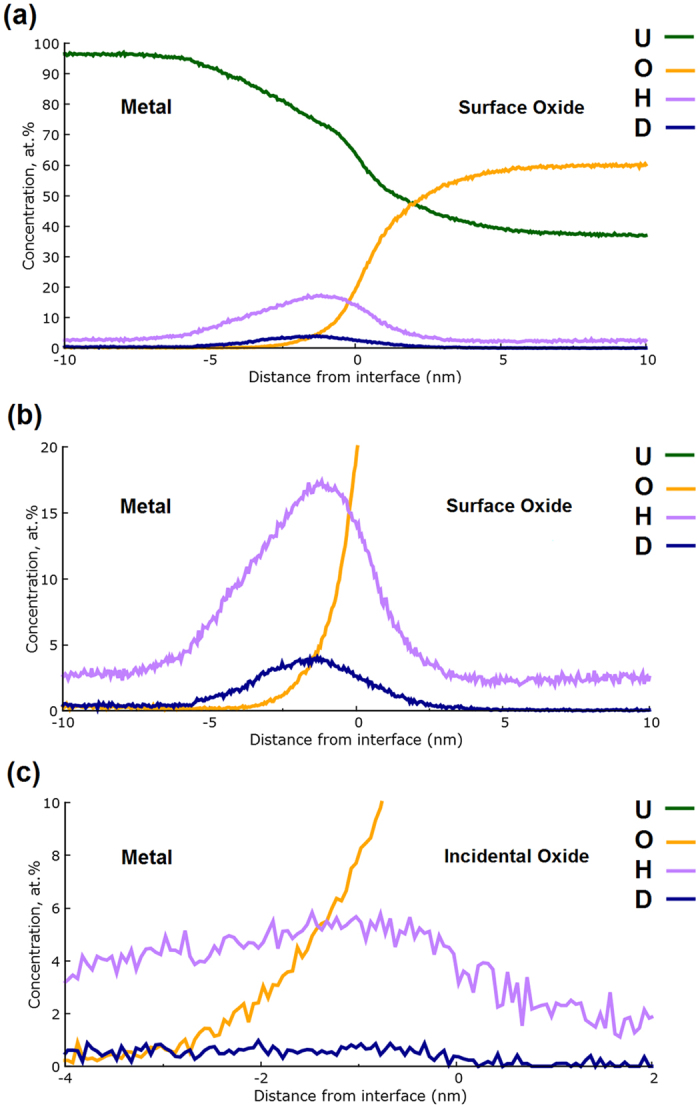
(**a**) Proxigram of the interface at the top of the sample (marked as ‘1’ in Fig. 5). (**b**) shows a close-up of the interfacial region (marked as ‘1’ in Fig. 5) where a clear deuterium signal mirrors the location of the hydride peak observed in Fig. 2. (c) shows a similar proxigram for the incidental oxide from the same dataset (identified as ‘2’ in Fig. 5), showing negligible deuterium content.

Figure 6 shows the proxigram, in complex ion form, for the surface oxide-metal interface marked 1 in Fig. 5(b). As before, the overall morphology following water vapour oxidation is the same; an underlying pure U matrix, followed by an uneven metal-oxide interface decorated by a spike in hydride content, with a surface formed oxide layer increasing rapidly in oxygen content from ~2.5 nm below the interface up to approximately 60 at.% oxygen through the remainder of the oxide to the surface.

**Figure 7:**
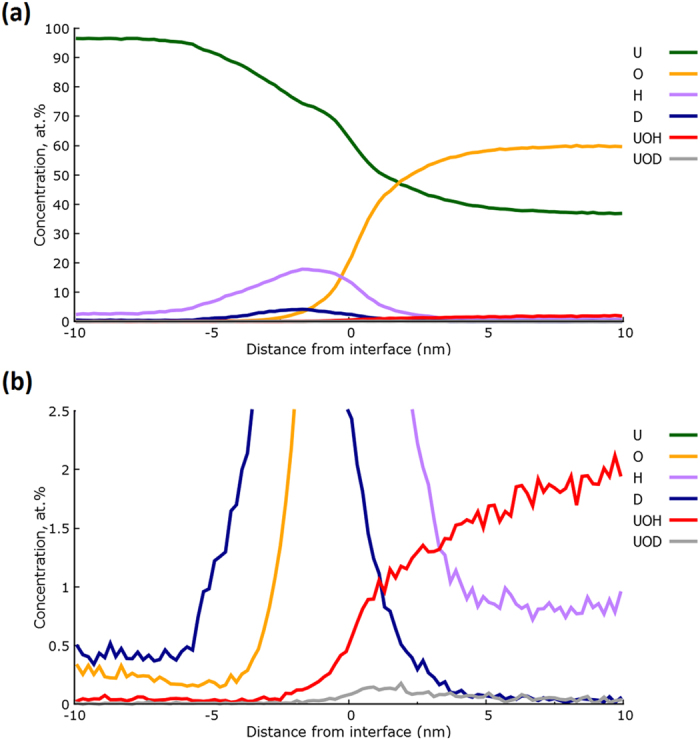
(**a**) Proxigram of the same interfacial region shown in Fig. 6(a,b) (marked as ‘1’ in Fig. 5), with the inclusion of UOH and UOD concentration levels. Figure 7(b) shows the same data magnified to better display the two hydroxide signals.

The UOH signal behaved similarly to that observed in the specimen exposed to ambient air, with little UOH at the oxide-metal interface, rising gradually to around 2 at.% within the oxide. The UOH signal is not shown in Fig. 6 as it obscures interpretation of the UD signal, but is represented for the same surface oxide proxigram in Fig. 7. A small quantity of approximately 0.2 at.% UOD was present at the interface, which fell to around half that value further into the oxide.

Reassuringly, the deuteride peak seen in Fig. 6(a) is not observed in the proxigram shown in Fig. 6(c) for the incidental oxide feature on the side of the tip, marked as ‘2’ in Fig. 5(b); demonstrated by the deuterium ion intensity being indistinguishable from the background noise levels detected by the experiment. This evidence confirms our earlier hypothesis that this incidental oxide forms after the intentional oxidation exposure, lift-out and needle preparation; and probably occurs during the transport of the specimens to the atom probe instrument.

Furthermore, the measured UD intensity for the interfacial region reduces to background levels at around 5.5 nm below the interface, whilst the equivalent UH content continues into the metal matrix at a near-constant level of around 3 at.%. The strong localisation of the UD feature is further evidence that the constant, non-zero UH plateau in the metal is an artefact due to hydrogen contamination occurring during tip evaporation (*i.e.* during data collection). Moreover, the localisation of the deuteride signal to the interfacial region supports the earlier hypothesis that the UH peaks observed in the ambient oxidation experiments reveal a significant aspect of the corrosion mechanism occurring for uranium oxidation in the presence of water vapour.

Clearly, the 120.0 Da and 240.0 Da peaks we ascribe to UD match the location of the UH layer observed at the interface in the ambient oxidation experiments extremely well. This result, confirmed by the comparison of proxigram data between Figs 2 and 6, provides irrefutable evidence that the hydride layer at the metal-oxide interface is a real chemical feature, rather than an artefact of hydrogen contamination from the vacuum system. The width of this UD layer was consistently 5 nm–8 nm in thickness for all specimens where the metal-oxide interface was successfully observed; thus, slightly wider than the peak observed in the ambient oxidation experiments. However, it is unclear from the data presented here whether this apparent increase in the hydride layer thickness when deuterated species are present is significant, since the conditions employed (i.e. air compared to D_2_O) cannot be considered as completely equivalent. However, we can confidently state that the thickness of this hydride layer cannot increase indefinitely as a function of oxidation, since previous SIMS investigations by the authors, and from the literature^6^, have failed to reveal the presence of hydride even when examining heavily oxidised samples.

The interfacial deuteride feature observed in Fig. 6 is accompanied by a corresponding hydride feature. Although this could be due to hydrogen contamination from the vacuum chamber, the relative concentrations of deuteride to hydride suggest several alternative explanations. This could be simple contamination of the D_2_O source used in the deuterated water vapour experiments, since its purity was not specifically confirmed, however it could also be a result of subsequent reaction with air, during transport between sample preparation and analysis. This could suggest that the hydride is being both consumed and regenerated as the reaction proceeds, and the observation of both deuteride and hydride within the same interfacial layer could reveal evidence of this continual regeneration process for the hydride layer.

The rate of uranium corrosion in water vapour environments is notably faster than that observed in dry oxygen or dry air, and the stoichiometry of the H_2_O-formed oxide shows less divergence from that of pure UO_2_^1^,^2^,^6^. In this investigation we have unambiguously demonstrated the formation of a thin hydride layer during water vapour corrosion, something which cannot possibly form in pure oxygen environments. This hydride layer has been demonstrated to reside at the oxide-metal interface and likely imparts a physical separation of the oxide from the underlying bulk metal. Thus, for oxidation of uranium in water vapour environments the presence of the hydride layer suggests that water performs a fundamental function within the reaction mechanism, and hence accelerates the reaction kinetics compared to O_2_ driven corrosion. The presence of hydroxyl content within the oxide, indicates that the hydroxyl ions are the oxygen source for the formation of additional uranium oxide, however further experiments using exposure from pure O_2_ in the absence of any humidity would be required to confirm this conclusion.

If hydroxyl ions provide the oxygen source atomic hydrogen will also be present as a free radical, which would readily establish a layer of hydride through reaction with the metal. As subsequent hydroxyl ions diffuse across the steadily expanding oxide layer, oxidation of the hydride must occur to generate new oxide at the hydride-oxide interface. This oxidation process would consume the hydride interfacial layer, and as our results suggests a near constant, albeit thin, hydride layer, this layer must undergo continual regeneration from the highly reactive atomic hydrogen produced during the decomposition of OH species at the oxide-hydride interface. The mechanism for the regeneration of the hydride layer is not clear from this APT study; however, the factors controlling the thickness of the hydride will be dependent on this regeneration mechanism, and are probably determined by the competitive rates for the reaction of hydrogen free radicals to either (i) form molecular hydrogen or (ii) regenerate the hydride.

A further consequence of a near constant thickness hydride layer being maintained at the oxide-metal interface is that, as the oxidation reaction proceeds, excess hydrogen must be created at the oxide-hydride interface, as evidenced from gas phase data in pure water vapour reaction studies^1^. The influence of this hydrogen continually flowing through the oxide, from the hydride-oxide interface to the surface, would be expected to maintain a reduced (*i.e.* certainly not hyper-stoichiometric) oxide during the water driven oxidation of uranium, which is consistent with both the stoichiometry of our oxide layer and the observations of Baker *et al*.^1^,^2^.

The atom probe experiments demonstrate clear evidence that the formation of hydride, whilst limited to a very thin interfacial region between the oxide and metal, occurs during the corrosion of uranium in the presence of water vapour. Without using such a high-resolution technique, detection of a layer only a few nanometres in thickness is incredibly challenging, yet it appears this hydride layer plays a pivotal role in the corrosion mechanism, and might explain why uranium corrodes faster in the presence of water than in oxygen alone. This finding, in addition to the novel corroborative technique of using deuterated water to prove that the hydride formed was a genuine feature, should lead to further advances in the study of actinide corrosion and the study of hydrogen using APT.

### Conclusions

APT has been successfully employed to study the composition, at the nanoscale, of the oxide which forms during the oxidation of uranium within water vapour containing environments. APT has offered unambiguous evidence, obtained by using both ambient and deuterated water sources, for the presence of uranium hydride being formed during the oxidation reaction. Moreover, this hydride is demonstrated to form specifically at the oxide-metal interface as a near-constant layer of 3 nm–5 nm in thickness.

The APT investigation performed demonstrates that the oxide stoichiometry is consistent for all samples measured, plateauing at ~60 at.% O; signifying a hypostoichiometric oxide not inconsistent with the literature. Furthermore, within the oxide APT reveals the presence of hydroxyl species (detected as OH and UO_x_H molecular ions), which suggests they play a significant role in the oxidation mechanism. In fact, previous hypotheses on the mechanism of oxidation for uranium in water vapour environments have required a technique which can determine the presence of either OH^−^ or O^2−^ species within the oxide. Our results support an oxidation mechanism driven via the diffusion of hydroxyl species. This is consistent with our finding of a hydride interfacial layer at the oxide-metal interface, since it is difficult to explain its presence for a mechanism based on O^2−^ diffusion.

Through the combination of the above findings, namely the near-constant thickness hydride layer at the oxide-metal interface and the oxidation mechanism being driven via the diffusion of hydroxyl species, we suggest that excess hydrogen must be generated at the hydride-oxide interface which would be expected to diffuse through the oxide to escape at the oxide surface. This is consistent with literature observations on the production of excess hydrogen during the water oxidation of uranium metal, but also offers mechanistic insight into the reason for the oxide not increasing in oxidation state and becoming hyperstoichiometric UO_2+x_, since the diffusion of (reducing) hydrogen species through the oxide would maintain the oxide at a reduced oxidation state, or reduced stoichiometry.

Due to the tendency for some hydrogen signal to be detected in atom probe from hydrogen-containing molecules in the steel-walled analysis chamber, a novel additional experiment was performed, by exposing the same material to deuterated water. The metal-oxide interface of the uranium metal exposed to D_2_O showed a clear peak of uranium deuteride, which was not observed either in the original H_2_O-exposed specimen, or in incidental oxides on the side of the D_2_O-exposed specimen formed after sample preparation. The presence of this thin deuteride layer proves that the source of the observed hydride peak is the water vapour, and that an intermediary step of uranium hydride forms as part of the mechanism of uranium corrosion, which had until now only been speculated.

The evidence presented here, using APT to study the microstructure of the oxide formed on uranium in water vapour environments, will vastly improve the construction of mechanistic oxidation models used to enable predictions of long-term safety of stored uranium materials.

## Methods

Coupons of high purity uranium 10 × 10 × 0.6 mm in dimension were polished using successively finer silicon carbide paper, 320grit, 600grit and 1200 grit, using distilled water as a lubricant. The sample was washed clean with HPLC methanol and dried in air. For the air exposure experiments firstly studied, the polished sample was exposed to air for 60 minutes before being placed in the focused ion beam (FIB) system. For the subsequent D_2_O experiments carried out to unambiguously determine the presence of deuterides (as proxies for hydrides) in the experiments, the polished coupon was placed in a room-temperature pressure vessel containing deuterated water for 2 hours.

Specimens were prepared by FIB on an FEI Helios NanoLab 600 instrument using a lift-out and polish method^14^. Sections of typical size 35 × 3 × 7 μm were extracted from the sample material using a Kleindeick micromanipulator in the FIB, before mounting onto silicon microtips. The mounted specimens were then sharpened into needle-shaped tips using annular ion milling. SEM images of the finalised tips showed tip radii of less than 70 nm. Following FIB preparation, the specimens were transported to the atom probe in an argon back-filled cell, aiming to minimize as much air exposure as possible between preparation and analysis. Bulk elemental analysis indicated carbon content to be <50 ppm, with other impurity elements <5 ppm.

**Figure 8:**
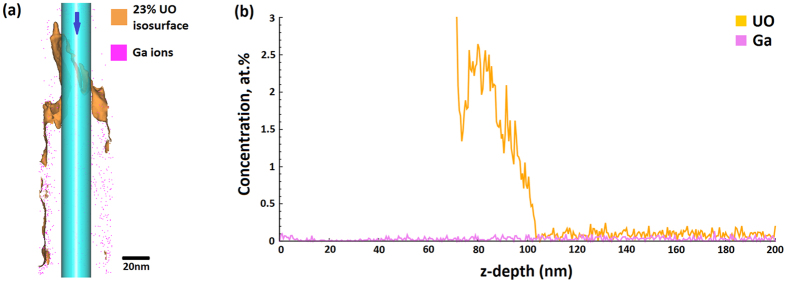
(**a**) Atom map showing the location of the Ga atoms (pink) compared to the position of the UO interface. A cylindrical region of interest has been taken through the centre of the dataset. The arrow indicates the direction taken for Fig. 8(b), a depth profile of Ga content through the z-direction of the cylindrical region of interest.

Typically, a layer of protective metal such as tungsten or platinum is deposited on the surface of a material prior to lift-out for protection against ion damage. In the case of the uranium it was found that the presence of a protective layer of Pt had a detrimental effect on the atom probe yield, as fracture could occur at the interface between the Pt and U. However, due to the large mass of the specimen atoms compared to that of the Ga^+^ ions, the damage from the ion beam was minimal, with Ga content measured with the atom probe at approximately 0.1 at.% even close to the surface. Figure 8(a) shows the distribution of Ga ions in the specimen, which are primarily located at the edge of the specimen. A cylindrical region of interest, also shown in Fig. 8(a), was used to create a depth profile of Ga implantation in a typical sample in the direction of the arrow shown. The Ga level is around 0.1 at.% at the very surface, and the concentration of Ga in the centre of the dataset is extremely low. The Ga peak at 69 Da is shown in Figure S.3 of the supplemental material, and is of significantly less intensity than any of the major peaks. Based on this experience, no protective layer was deemed necessary for the FIB preparation of the materials presented in this paper.

APT analysis was performed using a Cameca LEAP 3000X HR atom probe, using a pulsed green laser (532 nm) and a laser power of 0.5 nJ. The laser was set at a frequency of 160 kHz to prevent ‘wraparound’ of the heavy (and slow in terms of time-of-flight techniques) uranium atoms into the beginning of the next pulse. Specimens were cooled to 50 K and kept at a vacuum better than 3 × 10^−11^ Torr. The evaporation rate (the percentage of laser pulses in which an atom is detected) was varied between 0.1 and 0.5%.

## Additional Information

**How to cite this article**: Martin, T. L. *et al*. Atomic-scale Studies of Uranium Oxidation and Corrosion by Water Vapour. *Sci. Rep.* **6**, 25618; doi: 10.1038/srep25618 (2016).

© 2016 British Crown Copyright/AWE

## Supplementary Material

Supplementary Information
