# Supplementary material for: Atomic-scale Studies of Uranium Oxidation and Corrosion by Water Vapour
Source: Sci Rep. 2016 Jul 12;6:25618. doi: 10.1038/srep25618 (PMC4941397; doi:10.1038/srep25618)
Supplement: Supplementary Information [file srep25618-s1.doc]

**Atomic-scale Studies of Uranium Oxidation and Corrosion by Water Vapour**

T.L. Martin^1^*, C. Coe^2,3^, P.A.J. Bagot^1^, P. Morrall^2^, G.D.W Smith^1^, T. Scott^3^ and M.P. Moody^1^

1 University of Oxford, Department of Materials, Oxford, United Kingdom OX1 3PH

2 AWE, Aldermaston, Reading, Berkshire, United Kingdom, RG7 4PR

3 Interface Analysis Centre, University of Bristol, H.H. Wills Physics Laboratory, Bristol, United Kingdom, BS2 1TL

*Corresponding author: [tomas.martin@materials.ox.ac.uk](mailto:tomas.martin@materials.ox.ac.uk)

**Supplemental Information**

The three figures contained in this supplemental file show the mass spectra for the 1+, 2+ and 3+ charge states of the uranium isotopes and their complex ions for the key datasets used in the manuscript. The mass spectra for both ambient air-exposed and deuterated water-exposed specimens are shown to compare the differences in observed peaks between the two.


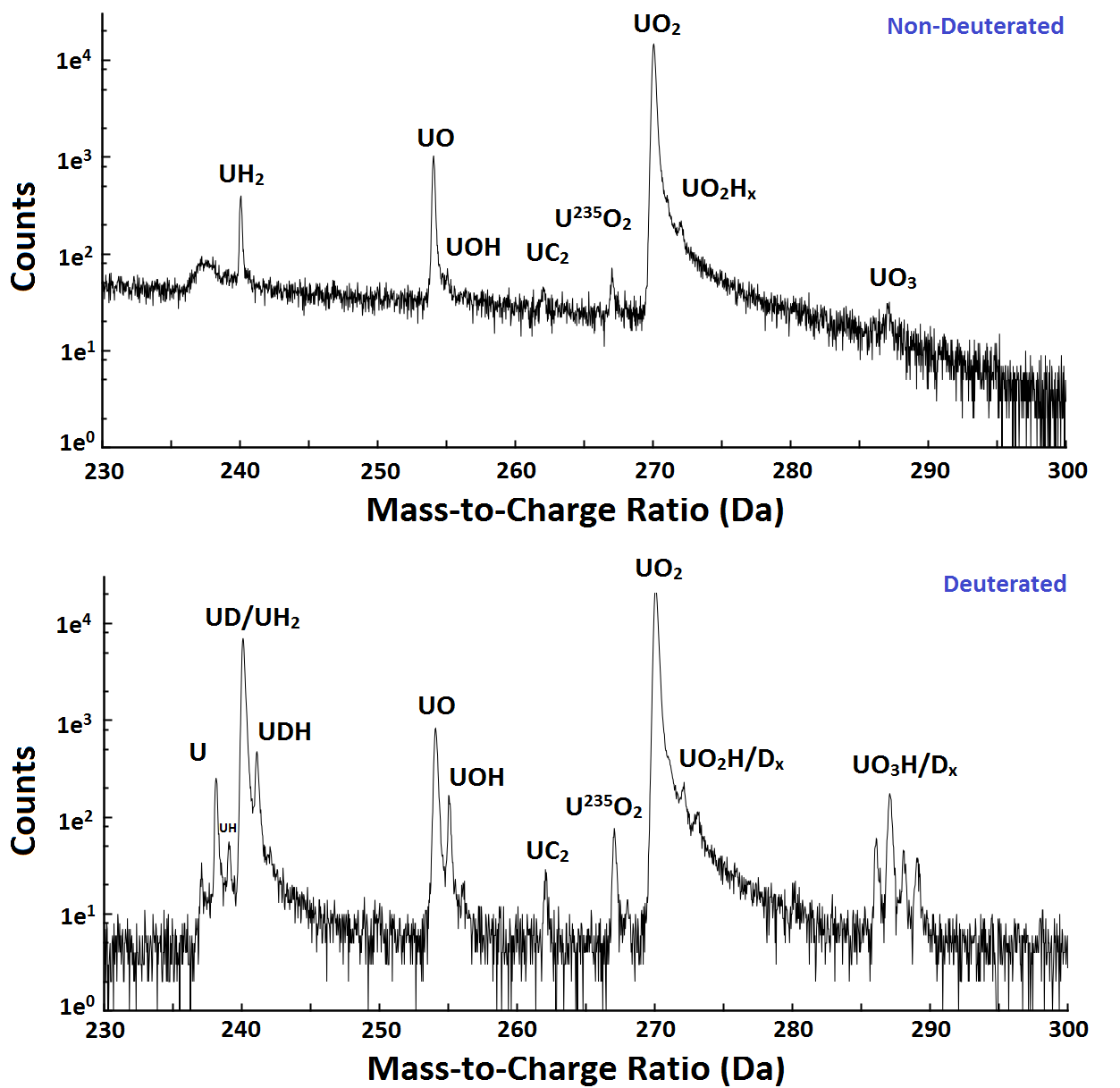


Figure S1: Mass spectrum of oxidised depleted uranium focused on the singular charged ionic states between 230 Da and 300 Da, comparing the behaviour from exposure to (a) ambient air and (b) deuterated water vapour


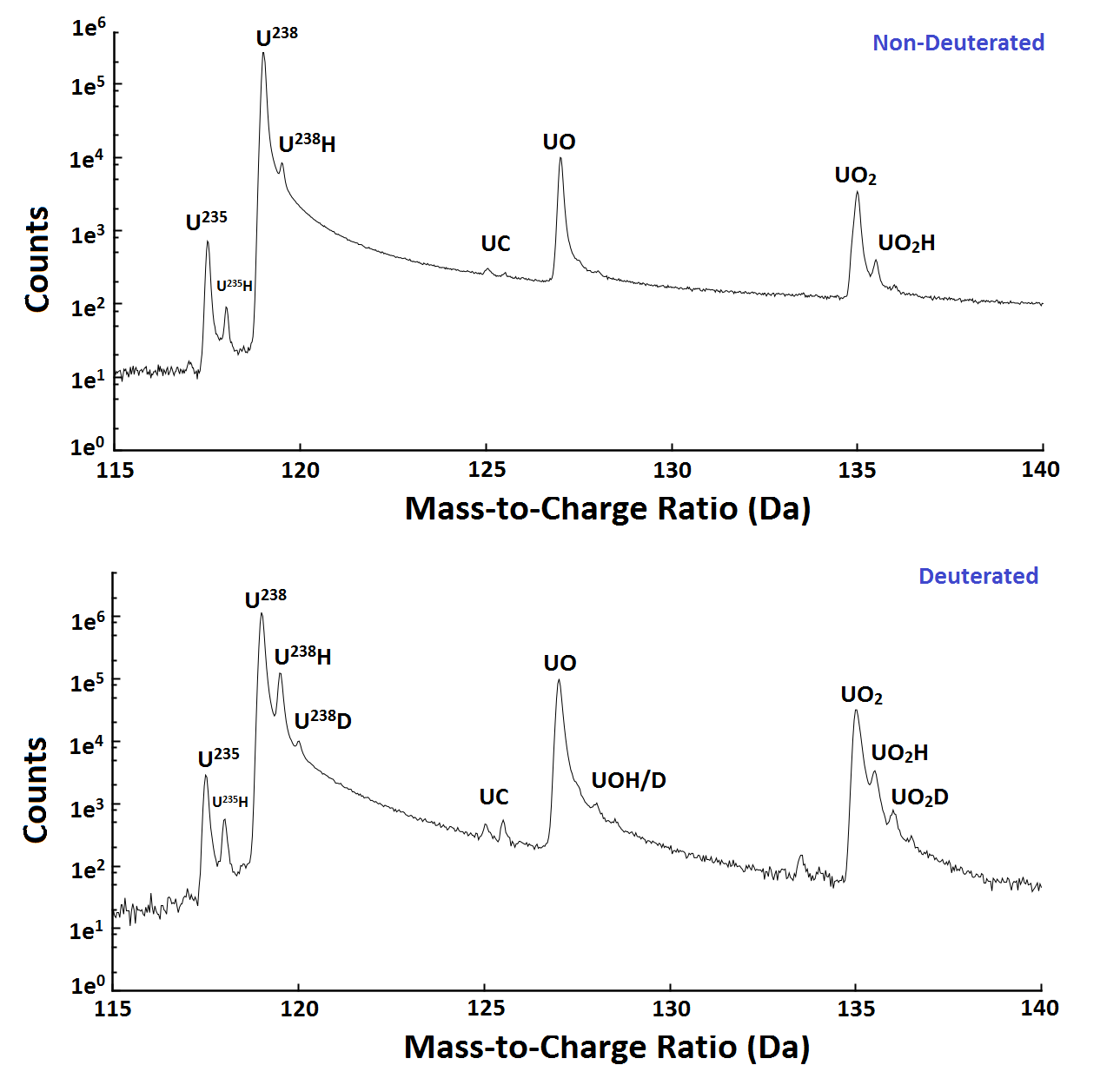


Figure S2: Mass spectrum of oxidised depleted uranium focused on the doubly charged ionic states located between 115 Da and 140 Da, comparing the behaviour from exposure to (a) ambient air and (b) deuterated water vapour


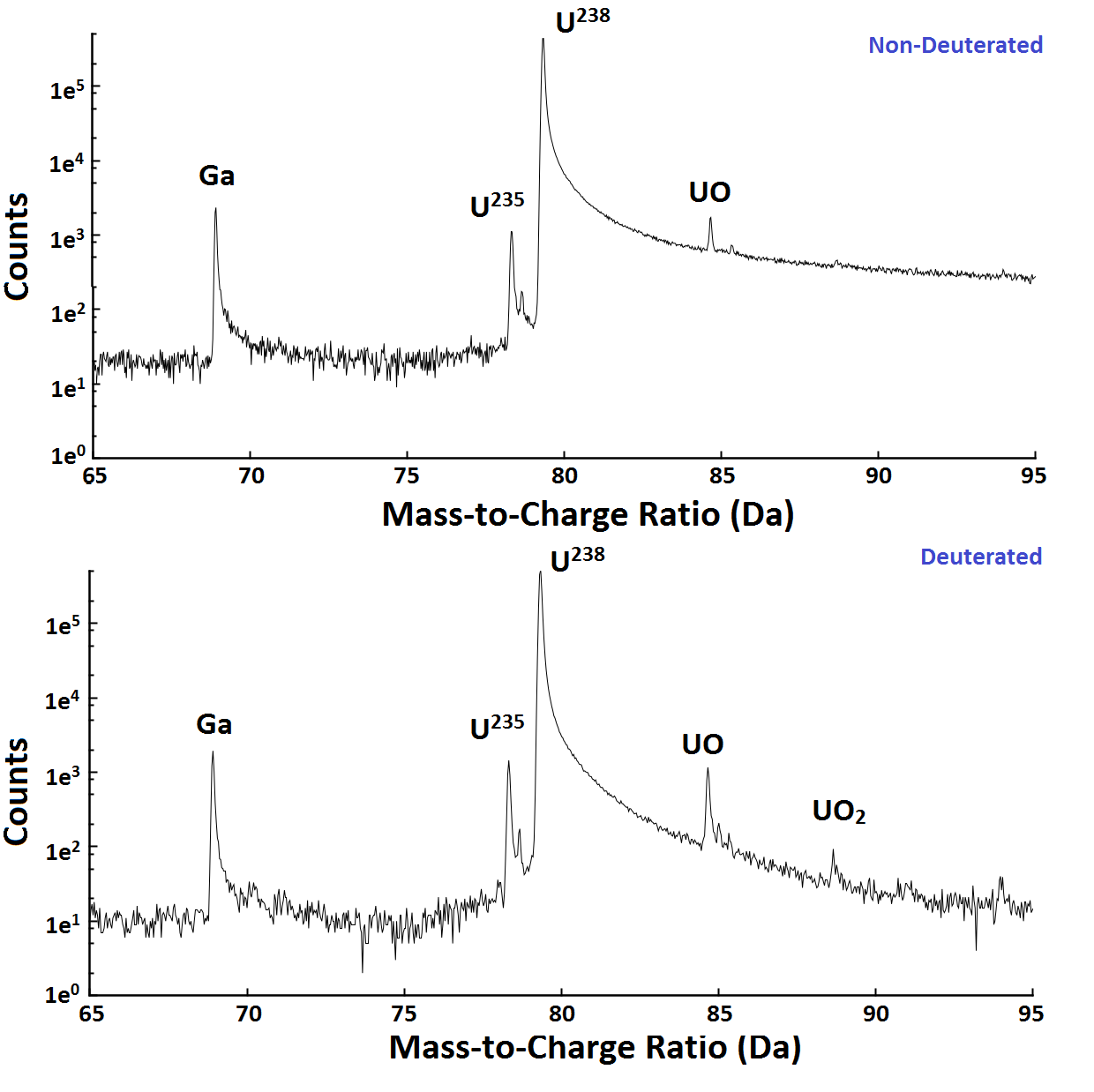


Figure S3: Mass spectrum of oxidised depleted uranium focused on the triply charged ionic states between 65 Da and 95 Da, comparing the behaviour from exposure to (a) ambient air and (b) deuterated water vapour. The Ga peak at 69 Da due to FIB preparation is also shown.
